# Mechanically Tunable Spongy Graphene/Cellulose Nanocrystals Hybrid Aerogel by Atmospheric Drying and Its Adsorption Applications

**DOI:** 10.3390/ma14205961

**Published:** 2021-10-11

**Authors:** Yuanzheng Luo, Zhicheng Ye, Shuai Liao, Fengxin Wang, Jianmei Shao

**Affiliations:** School of Electronic Information Engineering, Guangdong Ocean University, Zhanjiang 524088, China; luoyz@gdou.edu.cn (Y.L.); yezhicheng@stu.gdou.edu.cn (Z.Y.); liaoshuai@stu.gdou.edu.cn (S.L.); lanyu@stu.gdou.edu.cn (F.W.)

**Keywords:** graphene aerogel, cellulose nanocrystals, oil-absorption

## Abstract

For expanding applications of spongy graphene aerogels (GAs) cost-effectively, we report a marriage of the two-step hydrothermal reduction and atmospheric drying method to fabricate a spongy CNC-graphene aerogel (CNG) with oil/water selectivity and tunable mechanical strength by a low-cost and straightforward approach. The reduced graphene oxide (rGO) with CNC by the ice-templated method can give rise to forming the hierarchical structure of hybrid GAs within the PUS network. Meanwhile, the fractured structure of PUS with a pre-compressive step arouses more versatility and durability, involving its selective and high-volume absorbability (up to 143%). The enhanced elastic modulus and more significant swelling effect than pure sponge materials give it a high potential for durable wastewater treatment.

## 1. Introduction

Assembling graphene to a three-dimensional (3D) porous monolith while keeping the intrinsic advantage of the building blocks is of great promise to achieve the practical application [1,2]. Cellular graphene bulk material, commonly known as Graphene aerogel (GA), most approaches this nano-architectonic idea owing to its excellent electrical conductivity, large surface area, and low density [3]. Self-assembly during GO reduction process and freeze-casting of intermediate hydrogel are two main 3D assembled strategies that have been reported for “strong” graphene aerogel preparation. Liu et al. fabricated elastic graphene aerogels using directionally grown ice crystals as templates followed by freeze-drying [4]. The obtained anisotropic aerogel with a hierarchical porous structure could recover to its initial height after 20 times of axial compression and high oil absorption cycles. Dai et al. prepared an ultralight dual-network GA through a straightforward hydrothermal reduction followed by lyophilization [5], and the hierarchical porous network structure improved the absorption capacity toward various solvents, with absorption capacity (ratio of weight before and after soaking) up to 310 g/g. Gao et al. synthesized a spring-like 3D graphene aerogel with a long-range lamellar microstructure to undergo the large monolithic deformation by bidirectional freezing and freeze drying strategies [6]. To replace or clear the soft template of hydrogels, well-designed hierarchical structures of GAs are commonly formed by freeze-drying or a supercritical drying technique [7]. It is well known that these unique drying strategies enhance energy consumption and complicate the preparation process. Therefore, the ambient pressure drying (APD) method was employed to prepare compressive GAs by controlling the freeze rates of partially reduced GO (PRGO) and reduction degree of GO carefully [8,9]. For instance, Yang et al. [10] synthesised graphene aerogel by utilizing low-energy and low-cost atmospheric drying technology, which also endowed the compressive GAs with well-developed internal porosity and superelasticity (maximum strain of 93% for 3 cycles, 70% for 1000 cycles).

Additionally, 3D graphene/cellulose monoliths utilizing APD showed excellent hydrophobicity and elasticity, emerging as promising large-scale and low-cost absorbent materials in water treatment [11,12,13]. Xu et al. combined straightforward hydrothermal synthesis and low-lost ambient drying to prepare graphene/cellulose aerogel with high porosity [11]. Its adsorption capacity for various solvents is in the range of 87–207 g/g. Nevertheless, recent research found that the high-content nanocellulose sandwiched between graphene flakes can endow the hydrophobic aerogels with a higher modulus and larger oil storage interspace. Although this ultralow density endowed the cellulosic GAs with excellent mass-based absorption capability, the mechanical properties decreased with an increase of compression cycles, which are unfavorable for preparing large-scale macroscopic oil absorbents. Recent exploration of polymer/graphene materials is directly motivated by its possibility for potential oil application. These polymer matrices, polyurethanes (PU) foams with outstanding molecular design, could introduce elastic character and dimensional stability into the 3D graphene structure. Qiang et al. synthesized a Graphene/PUS composite via a straight dip-coating method [14], and the functionalized sponge coated with the graphene “skins” exhibited good water repellency.The spongy skeleton strategy has the potential to improve the robustness of the above-mentioned graphene-based absorbents. In addition, thermoplastic PU is notoriously difficult to recycle. Large quantities of waste PU sponges are generated from the packaging industry. Therefore, exploring the application of recyclable spongy graphene absorbent by a low-cost APD technique is a greener and more energy-efficient way to solve oil pollution.

Herein, porous cellulose graphene aerogel (CNG) was prepared using a two-step reduction process without lyophilization, where the cellular walls of aerogels enclosed by macroscopic-scale sponge porous scaffold led to a primarily enhanced mechanical performance. Even when the impact load with maximum (99%) strain is applied, the CNG exhibits resilience capacity without fracture and degradation. In addition, the cooperation of GAs and sponge porous cell walls endows a water droplet “sticky” hydrophobicity, which can absorb n-heptane over 1.3 times its volume and 120 times its weight and reuse manually about 1000 times. More importantly, these graphene sponges display a noticeable swelling effect, which means that adsorbed oil extended storage in more volume space of the nanocomposite. The CNG enabled a combination of visible polymer framework and microscale graphene scaffold, which can be successfully used for efficient and cost-effective water purification. These properties also demonstrate that, if graphene sheets are assembled into polymer skeletons where graphene nanosheets are kept, novel and combined properties will be observed.

## 2. Materials and Methods

The recycled PUS samples were obtained from Shenzhen Tenghuida Electronic auxiliary material Co., Ltd. The Cellulose Nanocrystals (CNCs) were obtained from ScienceK (http://sciencek.com/, accessed on January 2018) and its length nanoscale is approximately 100 to 200 nm size range, with the length–diameter ratio being 10–20. Graphite powder (CP), L-Ascorbic acid (AR), sulfuric acid (98% ), sodium hydroxide (AR), potassium permanganate (AR), and hydrogen peroxide (AR) were supplied by Shanghai Aladdin Bio-Chem (China) and used without further purification. Graphene oxide as a raw material was synthesized according to a modified Hummer’s method [15]. The radial length of the obtained two-dimensional GO sheets ranged from 0.1 to 20 μm. The 1.5 mg/mL CNC aqueous dispersion and GO suspension with different concentrations (3 mg/mL, 4 mg/mL, 5 mg/mL) were mixed for further processing. The pretreatment of the PU sponge was conducted through a strong pre-compression up to a certain strain and are cleaned with ethanol and placed in an oven to dry before use specifically, which is completed by adjusting the control distance of the 12-ton tablet press keeping it for 24 h PUS with a tetragonal or cylindric morphology dips coating of the GO/CNC mixture solution in a 100 mm culture dish. Straight after 30 min of ultrasonic dispersion, the mixture is a nearly homogenous dip-coating material. After the addition of 25 mg ascorbic acid in a 200 mL beaker, GO sheets are hydrothermally assembled for 2 h. The intermediate gel-like solid was obtained and then sequentially subjected to a wash in deionized water; this step was repeated about three times till the PH near neutral. Subsequently, a freeze casting process was carried out by the breaker partially submerged in an ethanol/dry ice cooling bath for 2 h. After further reduction, the reduced hydrogel was transferred to an air dry oven under ambient conditions for 12 h. Finally, the CNG-X materials were obtained after annealing treatment at $200{\;}^{\circ }$C for 2 h (where X denotes the concentration (mg/mL) of GO in the synthesis step). On the other hand, the same process was used to prepare the G/PUS product without mixing CNC suspension.

## 3. Characterization

CNG was characterized with a Nicolet 6700 Fourier transform infrared spectroscopy (FT-IR). Scanning electron microscopy (SEM) images were obtained by a Hitachi SU8010 at 20 kV. The X-ray diffraction (XRD) analysis is done with an X-ray source of CuKα radiation at a generator voltage of 40 kV and a generator current of 40 mA (X’Pert PRO DY2198). TEM observations were performed with a JEOL JEM-2100F TEM at 200 kV. The Young’s modulus of CNG was determined by a Zwick/Roell testing machine (Z010 TH) using a displacement-controlled compression mode with a rate of 5 mm/min. Contact angle measurements were conducted with a JC2000ACS drop shape analyzer (Zhongchen Digital Technology and Equipment, Shanghai, China). The precompression of the samples was conducted using a 12-ton tablet press (Shangha Tianqi Pharmaceutical Machinery Company, Shanghai, China).

## 4. Results

**Figure 1:**
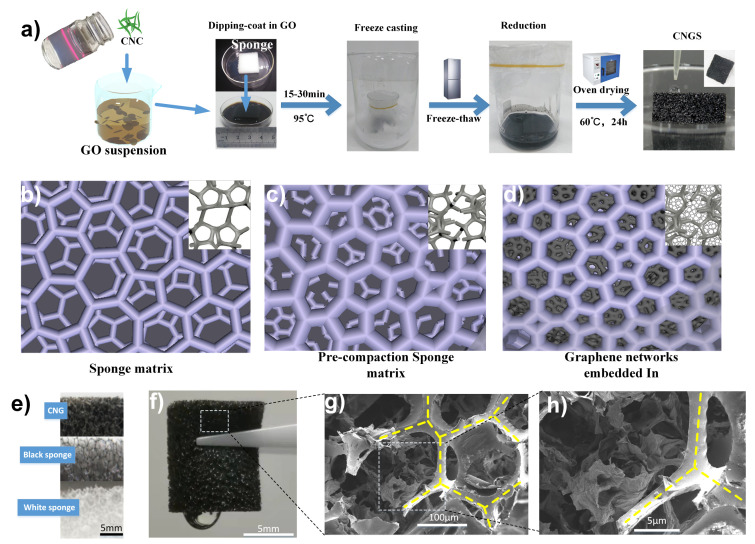
(**a**) Illustration of the preparing process of CNGs at ambient pressure condition. (**b**–**d**) the proposed schematic sketch for explaining the precompression and formation process of the cellular structure embedded in CNGs; (**e**–**f**) Digital images of CNG from macroscopic porous; (**g**–**h**) SEM images of porous CNG.

A high efficient and energy-saving wet shaping method under atmospheric pressure was demonstrated in this work. The typical fabrication procedure was depicted in Figure 1a); firstly, the CNC solution was obtained after an ultrasonic bath (the dispersible behavior of CNC proved by a Tyndall scattering effect). Then, the stable suspension containing CNCs and GO was achieved by mechanical and ultrasonic mixing, graphene oxide (GO) nanosheets in the solution as represented by brown sheets, and the green rod-like nanofiber represents wood-derived CNC. Then, the pre-compression of the waste PU sponge with the fractured network was utilized to integrate the microstructure of 3D graphene as shown in Figure 1b), and the rectangle PUS samples were immersed in as-prepared suspension. Notably, depending on the various shaped PUS samples, the proposed CNG could successfully fabricate the rectangle sample and other arbitrary shapes such as a cylinder. In addition, the CNC/GO/sponge mixture was partially reduced with ascorbic acid under a hydrothermal process, which is also the critical precursor of the high-performance graphene sponge preparation. After that, a 3D linked network forms between partially reduced graphene oxide (PRGO) and sponge backbones.

Moreover, these steps towards a more facile and green route, CNC/GA as an essential building block, are assembled into the 3D architecture of PUS. When the intermediate is frozen, GO nanosheets are expelled by the grown ice crystals, also known as ice templating, and for the formation of the micro-pore network in each sponge nest. The freezing temperature and the amount of oxygen-containing groups of GO are adjusted via reduction time [16]. These embedded networks of CNC/graphene sheets are formed among the void spaces of sponge bond and all networks along the macrocellular skeleton to yield an interconnected 3D compartmental micro-network. After ambient drying, the CNG with a hybrid porous structure is thus obtained. As a typical example, the digital photos of Figure 1e–f) show the difference between CNG and white/black PUS in macroscopic view by up-close photography. The opaque sponge porous array is characterized as regular hexagons with an average side length of 1 mm in each hexagon. The schematic microstructure images (Figure 1b–d) also show representative SEM images of the CNG (Figure 1g–h), which is presented that the isotropy sponge acted as a skeleton (marked in yellow dotted lines) for graphene nanosheets. A much better explanation is to treat these morphological features by adding the periodic sponge walls to the outlayer of the generated porous CNC/graphene. It is helpful to see how they are related macroscopically to the surface properties. The schematic diagram of a water drop in contact with the CNG demonstrated in Figure 1f). For a small droplet stick to the surface (the volume is 30 μL), the high adhesive force equals the weight. Thus, high hydrophobicity is expected, and yet the droplet sticks to the surface at the same time (Supplement Movie S1). The unique embedded multiple networks developed this hybrid porous and hydrophobic structure of CNG.

### 4.1. Chemical Evolution during the Reduction Process

**Figure 2:**
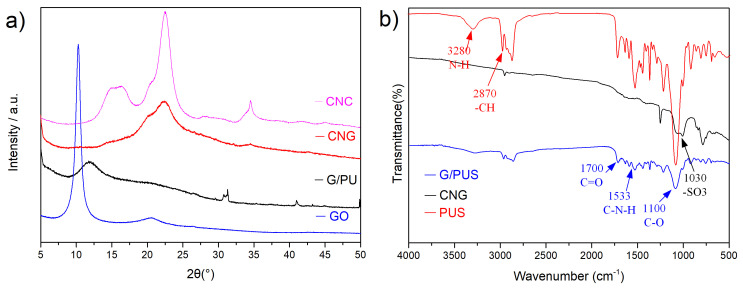
(**a**) XRD patterns of CNC, GO, G/PUS, CNG; and (**b**) FTIR spectrums of PUS, G/PUS, CNG.

The XRD diffraction patterns of CNC, the prepared GO and the rGO/PUS and after the CNC adding (CNG) are presented in Figure 2. A similar result of CNC exhibited characteristic diffraction peaks, which were in agreement with the diffraction peaks of cellulose [17]. The phases structural of the obtained GO present a sharp diffraction peak at 9–10${}^{\circ }$. In the XRD pattern of the G/PU, the characteristic peak of GO was not present, indicating the removal of oxygen-containing functional groups. Meanwhile, the broad diffraction peak at 2θ = 12.8° could be due to the PU’s crystallinity indicating its amorphous nature [18]. After adding the cellulose nanofiber, the XRD pattern of the CNG sample has the characteristic XRD peak of cellulose nanocrystal at 2θ = 22.6°. Compared with the high crystallinity of CNC, although the attenuation of the peak indicates a decrease in the degree of crystallinity, the original crystalline cellulose still maintained in the resultant [12]. The CNC succeeded in being loaded into a 3D graphene network via a 6h-hydrothermal process under ambient pressure drying. The typical FTIR spectrum (Figure 2b) was used in this study to identify the possible interactions between graphene, PU, and CNC. The polyurethane sponge contains carbonyl, amine, and amide groups, forming hydrogen bonds with graphene oxide during the complexation. The spectrum of PUS is clearly shown in Figure 2b), the strong peak centered at 3280, 1700, 1533, and 1100 cm${}^{-1}$ was attributed to the C-O, C-N-H, C=O and N-H. The spectra of G/PU presented the diminishing of the peaks, which have some similar chemical groups, indicating the involvement of these groups in the composite synthesis. After the CNC added, the spectra alterations for the CNG are the new bands at wavenumbers 1030 cm${}^{-1}$, showing the vibration of –SO${}_{3}$ groups. These sulfate groups are randomly distributed on the surface of cellulosic nanoparticles [17]. These changes indicated the successful implementation of introducing cellulose nanocrystals on the surface of CNG samples.

### 4.2. Tunable Morphology and Structure of CNG

**Figure 3:**
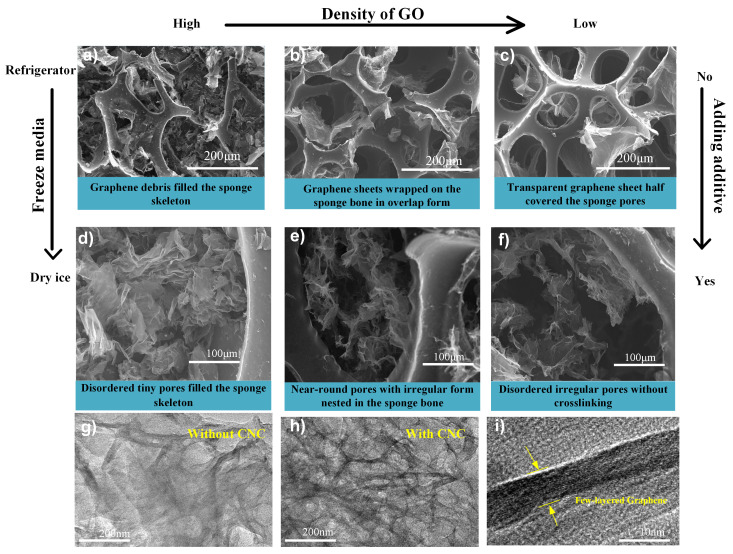
Microscopic structures of spongy CNC-graphene aerogels. (**a**–**f**) CNG prepared with different GO concentrations and freezing media exhibit distinguished structures; (**a**) 5 mg/mL, dry ice; (**b**) 4 mg/mL, dry ice; (**c**) 4 mg/mL, dry ice; (**d**) 5 mg/mL, refrigerator, adding CNC; (**e**) 4 mg/mL, refrigerator, adding CNC; (**f**) 3 mg/mL, refrigerator, adding CNC; (**g**–**i**): TEM images of the CNG prepared with dry ice as freezing medium.

Typical architectures of CNG sponge were prepared by the hydrothermal reduction and frozen by dry ice, as shown in Figure 1a. Most of the pores embedded in the sponge skeleton are nearly round, approximately the same as pure graphene aerogels. The macroscopic pores of PUS are visible to the naked eye, and the average diameter is a few hundred microns. The formation of embedded pores is associated with the environment, chemistry, and other factors. Thus, the hierarchical porous structure of the prepared CNGs can be tuned by different GO concentrations, freezing rates, and additives, which were investigated to realize successful morphology control. The illustrations in Figure 3 show the interior porous structure evolution with different parameters, including the shape, arrangement, and pore size.

Comparable results were obtained when different concentrations of the dispersed GO solution were tested. Figure 3a–c) shows graphene-sponge prepared by self-assembly of graphene sheets into the internal network of PU sponges without altering the original sponge morphology. However, no near-round graphene pores can be observed, with graphene sheets randomly assembling on the walls, displaying large disordered pores and damaged walls. Further improving the freezing media and adding CNC as an additive, porous graphene networks with near-round or disordered, irregular pores can be obtained. The graphene matrix induced by the soft templates are easy to rupture owing to the effect of capillary force, gravity, and surface roughness [19], and adding nanofiber or other one-dimensional nanomaterials in the freezing process can improve their stabilities. Additionally, the liquid–particle and particle–particle interactions during the dynamic freezing process also play a crucial role. The internal porous structure can be affected significantly by the freezing rate due to the ice formation path and style. Except for using dry ice as the rapid freezing medium, the refrigerator was utilized to freeze the CNC/GO aqueous dispersion relatively slowly to get a CNC/graphene-refrigerator sponge. The walls are thin and near-transparent, consisting of overlapped graphene sheets.These pores of slow-frozen graphene sponge are disordered with smooth walls. On the other hand, the SEM images in the next column (Figure 3d–f) show fluffy graphene sheet walls and disordered pores inherited from the ice template, which are identified with the yellow round marks. Notably, some tiny pores can be found at specific concentrations (5 mL/mL), preserving the morphology of ice crystals (denoted as CNG-5). Figure 3 also shows different magnification TEM images of the CNG-5 sample.The rod-like CNC acting as “spacers” between 2D graphene layers can efficiently impede their restacking and allow the microporosity of 3D graphene to survive ambient drying more easily, which is consistent with Figure 3g,h).The wrinkled nanosheets are transparent at the edge, indicating the presence of few-layers of graphene nanosheets. Therefore, the tunable freezing media and rate can change the porous structure made from an ambient pressure dring, then further changing the morphologies of the final graphene-based sponges.

### 4.3. Tunable Mechanical Strength of CNG

**Figure 4:**
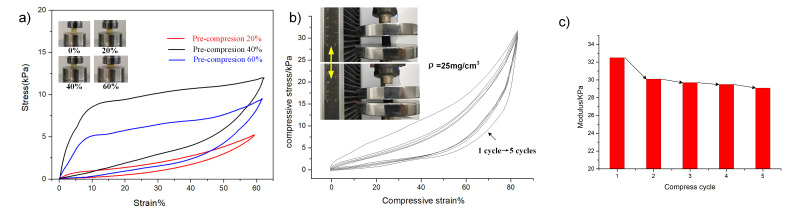
The stress–strain curves of pre-compressive PUS and CNG. (**a**) the stress–strain curves of PUS after preloading at different compressive strain(30%, 50%, 70%) for one cycle; (**b**) the stress–strain curves of the CNG at a strain of 80% for 10 cycles; (**c**) bar graphs of compressive modulus versus CNC content. Inset images are optical images of water droplets on the CNG.

Previous research has shown the mechanical properties of the pre-treated sponge relied mainly on the skeleton contact area and being verified that the fractured sponge network shows an increase of the contact area with the compressive stress in contrast to the network without pre-compression treatment [20]. Aside from the reinforcing effect of the organic skeleton, we also combined it with considering mechanical behaviors of embedded cellular graphene in static compression status. The axial compression tests of all samples were carried out using a universal testing machine (more details in Supplementary Movie S2). Figure 4a shows the photographs of the pre-compression step of the cylindrical PUS sample by high-strength steel fixtures on both sides. The stress–strain test of PUS was performed with a 100 N load cell kit and observed the variation of stress by different compression levels, as shown in Figure 4b. Although the organic skeleton of the samples increased the stress, a plateau stress region over a wide range of strain values appears. This feature means that the skeleton networks in the matrix do not affect the yield stress when the strain is between 7% and 45%, and the porosity values mainly determine the yield stress. Like the embedded graphene sheets in the PUS, the skeleton could enhance the compression strength of these graphene elastomers. Still, stress–strain curves revealed a smooth plateau region that deformation over this region occurs in a nearly constant state [12].

In contrast to what is observed in the CNG specimens, the micropores structure strongly affects the stress–strain response without a plateau stress region such as deformation over the above strain region. This phenomenon can be attributed to the high modulus of whisker-like CNCs nanoparticles and changes induced by the dual network, which combined polymer networks and graphene cellular porosity. The previous research also revealed that the compressive yield stress of porous graphene materials increases linearly with an increase in porosity [21]. In order to investigate the reusability, the cyclic axial compression of CNG-5 was measured. As shown in Figure 4b, the compressive-recovery curves exhibited its good resilience. In the first load—unload cycle, the stress linearly increased, demonstrating elastic strain changes larger than 60%. The elastic deformation of the polymer networks provides the initial compressive strength, bending, and shearing deformation of the graphene sheets further hindering the deformation under external force as the strain increased. The second cycle revealed many contractions; moreover, the typical hysteresis loops show a slight change in the following five cycles. As cycling continues, the loading-unloading path is repeated (indicated by arrows), and no flat stress regions occur in the stress–strain curves. With an increase of unload/reload loops, the modulus of CNG-5 decreases gradually and the compressibility tends to be stable (Figure 4c). Most graphene hybrid composites show the similar compressive properties after the residual stress is released [22,23].

### 4.4. Absorption and Swelling Properties of CNG

Owing to the hydrophobic porous structure and ultra-lightweight, the oil adsorption capacity (the ratio between the final and initial weight after absorption) of cellulose/graphene aerogels up to 198 times their weight [24]. Pristine CNG was weighed before it was entirely immersed into various organic solutions for the absorption rate study. The whole absorption process was fast and generally reached saturation within 5–10 s (precise time as shown in Supplementary Movie S3). Then, take out the soaked sample and stand for a period of time before weighing its weight. Saturated absorption capacities of absorbents are classified into two categories, weight gain and volume gain, depending on different application requirements. The oil-collection weight gain Q${}_{m}$ was calculated from the ratio of the mass of the absorbed oil M${}_{o}$ to the mass of the absorbing material M${}_{m}$ (Q${}_{m}$ = M${}_{o}$/M${}_{m}$). The volume-based absorption capacity Q${}_{v}$ is given by the equation Q${}_{v}$ = V${}_{o}$/V${}_{m}$, where V${}_{o}$ and V${}_{m}$ are the volumes of the absorbed oil and absorbing material, respectively. The process of oil adsorption is repeated at intervals of a period (30 s). The adsorbed oil can be recovered by simple mechanical squeezing for repetitive uses. Although the initial weight of CNG is roughly 20 times to these pure aerogels, it demonstrates high absorption capacities of 50 to 80 times their weight for the most common organic solvent.

**Figure 5:**
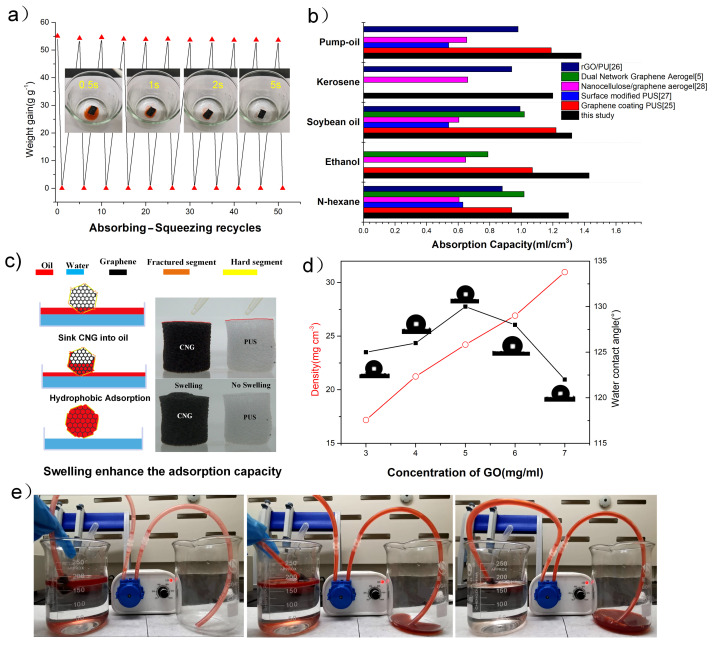
(**a**) Recyclability of CNG for absorption of n-heptane. Inset is the digital images during the process of removing oil on water surface, 1.3 mL pump-oil was stained with Sudan Red; (**b**) comparison of volumic adsorption capacities of different organic liquids (Graphene coating PUS, Surface modified PUS, Nanocellulose/graphene aerogel, dual Graphene Aerogel, rGO-PUS); (**c**) a schematic description of swelling effect during the process of removing oil on water surface; (**d**) the relationship between the GO concentration of density and water contact angle is measured; (**e**) the continuous adsorption experiment of n-heptane using a peristaltic pump.

In particular, the volume ratio of absorbed liquid is much higher than lightweight aerogel and other graphene-based foams as shown in Figure 5a), such as the nanofiber hybrid graphene aerogels [12], Graphene coating PUS [25,26], Surface modified PUS [27], and functional graphene sponge [5,28]. These volumetric oil absorption rates can be calculated according to the density of oil absorption material $\rho_{m}$ in the literature, due to the Q${}_{v}$ = V${}_{o}$/V${}_{m}$ = (M${}_{o}$$\rho_{m}$)/(M${}_{m}$$\rho_{o}$), the $\rho_{o}$ is a given condition. The absorption capacity Q${}_{m}$ = M${}_{o}$/M${}_{m}$ of these materials toward low-density organic solution such as n-heptane, n-hexane, ethanol were around 25–100 g/g, while, for higher density oil/organic solvents, such as diesel, mineral oil, and soybean oil, ranging from ∼100 to ∼160 g/g. Notably, the adsorption capacity exceeds 1 ml per cubic centimeter of CNG, which can also be interpreted as the swelling property of CNG after the adsorption of organic liquids. A schematic diagram for the typical swelling process is illustrated in Figure 5c). The adsorbed organic solvent enters into the hierarchical network and leads to an interlayer swelling effect occurring [29]. The swelling of the CNG increases their internal volume and interlayer distance largely, increasing oil storage space. In addition, the distinct volume ratio of various organic solvents suggests that the volumetric oil absorption rates can be altered by adsorbed organic solution and up to 142% as shown in Figure 5b). The experimental results show clear dependence variation of such swelling effect on the absorbed organic liquid, especially on the molecular weight of oil. The swelling ratio of CNG is significantly higher than neat sponge after adsorption of 0.2 mL ethanol (more details in Supplementary Movie S4). As shown in Figure 5d), the surface contact angle of CNGs prepared by different concentration GO (3–7 mg/mL) was 124.1°, 125.9°, 130.0°, 127.8°, and 122.2°, respectively. Therefore, the CNG-5 possesses higher hydrophobicity, which is beneficial for selective uptake of oils and organic solvents. Owing to the attractive properties of directional and selective absorption, the pumping experiment in an oil–water mixture was conducted by joining the pipe end with a cylinder CNG as shown in Figure 5e).The 50 mL n-heptane solution stained with Sudan red IV floating on the surface of water was removed by continuous pumping. The water level in the beaker was unaffected when the absorbent submerged under the water (as shown in Supplementary Movie S5). A comparison of the adsorption capacity and mechanical performance of different oil adsorbents is given in Table 1. These results demonstrate that CNG is a promising candidate for the selective adsorption of waste oils from water in practical applications.

## 5. Conclusions

In summary, spongy CNC/graphene aerogels have been successfully prepared via an ambient-drying method with a pre-compaction treatment. The corresponding hybrid structure allows CNGs to have unique compressive strength to afford compression cycling numbers and maintain a linearly mechanical property. Moreover, optimizing the freezing and drying process and controlling the internal microstructure with hierarchical pores simultaneously led to a high volumic oil-absorption capacity (up to 142%). The composite developed here also demonstrated a swelling effect with easy recyclability. Significantly, this energy saving synthesis process would be a key progress for realizing large-scale commercial production, and this graphene-based hybrid material provided access to assembling a monolith by embedding porous structures of graphene aerogel inside another porosint. The networks’ interactions changed the material’s bulk properties to find broader promising applications for graphene aerogels, including pressure sensors and petroleum contamination remediation.

## Figures and Tables

**Table 1:** Comparison of different oil adsorbents from past studies.

Adsorbent	Q (g·g${}^{-1}$)	Solvent	Recycle	Mechanicalness	Ref
PDMS/GO	6	Soybean oil	10	60% (50 cycles, 42.5 kPa)	[30]
FGN/PU	26	N-hexane	50	71.4% (400 cycles, 15 kPa)	[31]
Silk/G-sponge	53	Soybean oil	10	60% (100 cycles, 14 kPa)	[32]
Polyimide GA	25	Ethanol oil	10	60% (100 cycles, 2.3 kPa)	[24]
CNG	55	N-hexane	50	60% (100 cycles, 32.5 kPa)	this work

## Data Availability

The data presented in this study are available on request from the corresponding author.

## Supplementary Materials

The following are available online at https://www.mdpi.com/article/10.3390/ma14205961/s1, Movie S1: Inverted droplets, Movie S2: Axial compression test, Movie S3: Static oil absorption, Movie S4: Swelling effect, Movie S5: Pumping experiment.

## Abbreviations

The following abbreviations are used in this manuscript:

rGO	reduced Graphene Oxide
GA	Graphene Aerogel
CNG	CNC-Graphene aerogel
PUS	Polyurethane Sponge
PDMS	Polydimethylsiloxane
FGN/PU	functionalized graphene/polyurethane
